# Improve Dentin Bonding Performance Using a Hydrolytically Stable, Ether-Based Primer

**DOI:** 10.3390/jfb13030128

**Published:** 2022-08-26

**Authors:** Xiaohong Wang, Shinobu Yamauchi, Jirun Sun

**Affiliations:** 1 American Dental Association Science & Research Institute, Gaithersburg, MD 20899, USA; yamauchis0506@gmail.com; 2 Research Center for Electron Photon Science, Tohoku University, Sendai 982-0826, Japan; 3 The Forsyth Institute, Cambridge, MA 02142, USA; jsun@forsyth.org; 4 Harvard School of Dental Medicine, Boston, MA 02115, USA

**Keywords:** dental adhesive, bonding primer, hydrolytically stable, shear bond strength, micro-tensile bond strength

## Abstract

The objective of this study is to replace a traditional methacrylate-based primer (glycine, N-(2-hydroxy-3-(2-methyl-1-oxo-2-propenyl)propyl)-N-(4-methylphenyl) monosodium salt, NTG-GMA) with a hydrolytically stable ether-based primer (glycine, N-2-hydroxy-3-(4-vinylbenzyloxy)-propyl-N-(4-methylphenyl), monosodium salt, NTG-VBGE). The performance and durability of bonding composites to detin of two primers combined with methacrylate-based or ether-based adhesives were evaluated using shear bond strength (SBS) and micro-tensile bond strength (μTBS) combined with thermal cycling. The hydrolysis resistance of NTG-VBGE against hydrolysis was tested by challenging primed hydroxyapatite crystals with an esterase. The hydrophilicity of the primers and the resin spreading kinetics of adhesives on primed dentin were characterized by water contact angle measurements. The new primer NTG-VBGE was found to be compatible with both methacrylate-based adhesives and ether-based adhesives. The highest μTBS values were found in the test group of NTG-VBGE and ether-based adhesive, which was consistent with the resin spreading kinetics results. The more hydrophobic and hydrolytically stable primer/adhesive achieved improved dentin infiltration and bonding strength, suggesting significant potential for further developing dental restorative materials with extended service life.

## 1. Introduction

A dental bonding system bonds restorative composites to teeth and provides resistance to the separation of an adherend substrate (e.g., enamel, dentin, composites), distributes stress along the bonding interface, and protects the restoration’s interface against the penetration of esterases and bacteria in the oral environment that may cause secondary caries [1,2,3,4]. A typical dental bonding system includes etchants, primers, solvents, resin monomers, initiators, reinforcing fillers, and sometimes other functional ingredients such as antimicrobial agents [5,6,7]. Methacrylate-based resins are the most popular contemporary dental primers and adhesives for direct restorations [8,9,10]. Typical adhesive monomer mixtures contain 2-bis(4-(2-hydroxy-3-methacryl-oxypropoxy)-phenyl)-propane) (Bis-GMA) as a base monomer and 2-hydroxyethyl-methacrylate (HEMA) as a diluent monomer to enhance the handling properties and achieve suitable dentin infiltration [11]. However, the bonding durability and tooth-protection capability are frequently compromised by the failure of the tooth/restoration interface [12,13,14]. These failures are attributed to the hydrolysis-prone ester functional groups in the resin’s constituent monomers that are degraded by enzymes and cariogenic bacteria, incomplete infiltration of the dentin by the resin, and the high water sorption of the resulting resin network [15,16,17,18].

**Figure 1:**
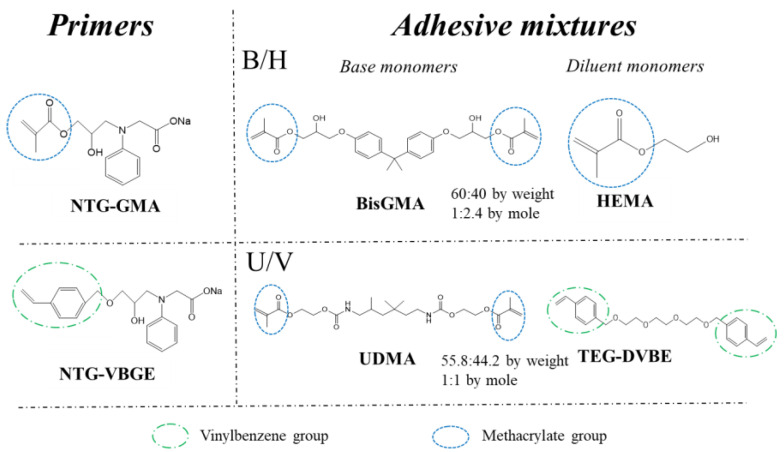
The chemical structures of monomers used in this study.

It is believed that high-performance dental bonding can be achieved by forming chemical bonds between a primer and either the inorganic or the organic components of a tooth, in particular, the Ca^2+^ within the hydroxyapatite mineral phases of the tooth [19]. Phosphate and carboxylic groups form chelation bonds with Ca^2+^ and, therefore, are expected to form such bonds during dentin priming, as has been found with carboxylate-based cements along with a bonding mechanism by amino-carboxylate functional groups [20]. On the same molecule, there should be a polymerizable functional group, such as methacrylate, that can react with the resin adhesive and thereby form a chemical bond between the adhesive and dentin [20]. A typical primer molecule, N-2-acetic acid-N-3-(2-hydroxy-1-methacryloxy)propyl-4-methylanaline sodium salt (NTG-GMA, structure shown in Figure 1), is one of the most studied compounds used in bonding formulations [21].

New monomers and polymerization mechanisms have been suggested to replace the hydrolyzable monomers such as Bis-GMA [22,23,24,25,26,27], to enhance hydrolytic stability by strengthening the network [28,29] and to reduce leachables [30,31]. Previously, we reported a hydrolytically stable, ether-based monomer triethylene glycol divinylbenzyl ether (TEG-DVBE) and primer glycine, *N*-2-hydroxy-3-(4-vinylbenzyloxy)-propyl-N-(4-methylphenyl), monosodium salt (NTG-VBGE) [22]. Strong and durable dental adhesives [32] and composites [33] have been developed based on TEG-DVBE. This study aimed to compare the bonding performance of the new ether-based primer NTG-VBGE with the traditional primer NTG-GMA. We hypothesize that: (1) NTG-VBGE is more hydrophobic and more hydrolytically stable than NTG-DMA; (2) NTG-VBGE can achieve strong dentin bonding with either traditional methacrylate-based or new ether-based adhesives; and (3) NTG-VBGE can facilitate a better dentin infiltration for ether-based adhesives.

## 2. Materials and Methods

### 2.1. Materials

Dental resin monomers, 2-bis(4-(2-hydroxy-3-methacryl-oxypropoxy)-phenyl)-propane) (Bis-GMA), urethane dimethacrylate (UDMA), 2-hydroxyethyl-methacrylate (HEMA), and glycine, N-(2-hydroxy-3-(2-methyl-1-oxo-2-propenyl)propyl)-N-(4-methylphenyl), monosodium salt (NTG-GMA), were provided by Esstech Inc. (Essington, PA, USA). Glycine, N-2-hydroxy-3-(4-vinylbenzyloxy)-propyl-N-(4-methylphenyl), monosodium salt (NTG-VBGE), and triethylene glycol divinylbenzyl ether (TEG-DVBE) were synthesized and fully characterized in our laboratory as previously reported [22]. The restoration composite used was Filtek™ Z250 Universal Restorative (Z250, 3M ESPE, St. Paul, MN, USA).

### 2.2. Preparing Dental Adhesives and Primers

Dental primers and adhesives were prepared by mixing resin monomers and photo-initiators according to compositions described in Table S1. The ester-based adhesive B/H was prepared by mixing Bis-GMA (base monomer, high viscosity) and HEMA (diluent monomer, low viscosity) at a mass ratio of 60/40 (mole ratio: 1.0/2.4). The ether-based adhesive U/V was prepared by mixing UDMA (base monomer, high viscosity) and TEG-DVBE (diluent monomer, low viscosity) at an equimolar ratio (mass ratio: 55.8/44.2). The initiator system comprised 0.2 wt% camphorquinone (CQ; Aldrich, Saint Louis, MO, USA) and 0.8 wt% ethyl 4-N, N-dimethylaminobenzoate (4EDMAB; Aldrich, Saint Louis, MO, USA). A Bis-GMA/HEMA-based dental adhesive, Adper™ Scotchbond™ Multi-Purpose Adhesive System (Scotchbond, 3M ESPE, St.Paul, MN, USA), was used as the commercial control and was applied following the manufacturer’s instructions. The chemical structures of the base monomers and diluent monomers as well as two primers utilized are shown in Figure 1. The primer “NTG-GMA” contains 5 wt% NTG-GMA and 20 wt% PMGDM + HEMA (1:1 by weight, and 0.2 wt% CQ + 0.8 wt% 4EDMAB to the weight of PMGDM + HEMA) acetone solution. While the other primer “NTG-VBGE” replaced the ester-based NTG-GMA with ether-based NTG-VBGE in the formulation.

### 2.3. Dentin Bonding Procedures and Shear Bond Strength (SBS) Evaluation

Human teeth used in all the tests were obtained according to the protocols approved by the American Dental Association (ADA)’s Institutional Review Board. The freshly extracted caries-free permanent molars were treated with 0.02% (*w*/*v*) sodium azide solution and stored in deionized water at 4 °C until use. Only molars that were extracted within 3 months were used. The SBS tests were carried out using a protocol previously established in our group [32]. The procedures of tooth embedding, 3-step bonding, and SBS evaluation are illustrated in Figures S1 and S2. Four groups of different combinations of two primers and two adhesives were tested: (1) NTG-GMA + B/H; (2) NTG-GMA + U/V; (3) NTG-VBGE + B/H; and (4) NTG-VBGE + U/V. 

Teeth were embedded with Fastray composite (Harry J. Bosworth Company, Skokie, IL, USA) in cylindrical holders and ground perpendicular to their long axis with 400-grit SiC paper until the occlusal enamel was completely removed. A three-step adhesive procedure entailed: (1) etching of dentin surface with a 32 wt% phosphoric acid gel (Scotchbond™ Universal Etchant; 3M ESPE., Seefeld, Germany) for 15 s and rinsing with distilled water (after rinsing, dentin surface was kept hydrated with a moist blotting paper); (2) applying primer by brushing on the dentin surface, accumulating 5 layers (only one layer was applied for the commercial Scotchbond Primer based on the manufacturer’s recommendations), air drying between layers to evaporate the solvent; then (3) applying bonding agents by brushing once on the primed dentin surface. The entire dentin surface was then light-cured for 10 s with the use of an 8 mm tip on a quartz halogen light source having 550 mW/cm^2^ intensity (Spectrum 800, Caulk/Dentsply, Milford, DE, USA). The intensity of the irradiation is measured by using a commercial laser power meter (PowerMax-USB PM2, Coherent Inc., Santa Clara, CA, USA). A poly(tetrafluoroethylene)-covered stainless steel ring (opening diameter = 4 mm; thickness = 1.5 mm) defined the bonding area through which the composite was applied onto the coated dentin. The ring was held down by a polycarbonate holder, and the iris was filled with the Z250 composite. The entire assembly was placed in distilled water for 4 min after 1-min light irradiation and stored for 24 h at room temperature before conducting a bond test in the shear mode. The ring and the composite were sheared off, at a crosshead speed of 0.5 mm/min, with a flat chisel pressing against the edge of the steel iris. The flat chisel was controlled by a Universal Testing Machine (Instron 5500R, Instron Corp., Canton, MA, USA). The maximum load was converted into the SBS by following this relationship, SBS = maximum load/area, where the area is the contacting area between composites and adhesives defined by the inside diameter (4 mm) of the steel ring. The mean values of SBS were the average of five measurements for each composition. 

### 2.4. Micro-Tensile Bond Strength (μTBS) and Thermal Cycling (TC) for Durability Evaluation

Teeth were embedded with Fastray composite (Harry J. Bosworth Company, Skokie, IL, USA) in metal cubic holders (Figure S3), then ground perpendicular to their long axis with 400-grit SiC paper until the occlusal enamel was completely removed. The bonding procedures were the same as described in SBS tests. After applying the bonding agent, Z250 composite was added in four 1 mm thick increments. Specimens were light-cured for 1 min using a quartz halogen light source at 550 mW/cm^2^ (Spectrum 800, Caulk/Dentsply, Milford, DE, USA) and then stored in distilled water for 24 h. Following the water storage, specimens were cut in the mesiodistal direction and parallel to the horizontal plane of the teeth using a 0.2 mm diamond disk (IsoMet^TM^ Diamond Wafering Blades, Buehler, Lake Bluff, IL, USA) at 100 rpm speed under running water. The resulting 0.7-1.0 mm thick beams were divided into two groups: one group was examined immediately after sectioning, and the other group was assessed after TC. The beams were glued to a μTBS testing device using Zapit (Dental Ventures of America, Corona, CA, USA). Tests were performed by using a Universal Testing Machine (Instron 5500R, Instron Corp., Canton, MA, USA) under tension at 1 mm/min until failure. The μTBS values (MPa) were calculated by dividing the load at failure by the cross-sectional bonding area, and they are reported as an average of 15 measurements using beams from three different teeth.

The TC was completed after the teeth were sectioned into beams. The beams for each test (each resin composition before or after TC) originated from 3–4 different teeth. TC tests were performed between 5 °C and 55 °C on beams prepared for μTBS tests using a device developed in our research center [34]. The temperatures were maintained by two water tanks. Specimens were switched between tanks with the dwell time in each tank for 30 s. The transferring time between tanks was 10 s. After the completion of 10,000 cycles, the average μTBS of each adhesive was determined and compared with the μTBS value before TC. The μTBS values were reported as an average of 15 measurements using beams from at least three different teeth. 

### 2.5. Hydroxyapatite (HA) Pellets as Model Surfaces to Study the Hydrophilicity and Biostability of Primers

HA crystals used in this study were square pellets (10 mm × 10 mm × 2 mm, APP-100, HOYA Technosurgical, Tokyo, Japan) with flat and smooth surfaces. The surface of HA crystal is very flat and smooth, with a surface roughness R_a_ = 5.2 ± 0.4 nm within a 5 μm × 5 μm scanning area measured by an atomic force microscope. HA pellets were washed with ethanol immediately before use, rinsed with distilled water, and dried by nitrogen. The surface modification of the pellets was carried out by immersing the cleaned pellets in 10 mL of 5wt% primer (NTG-GMA or NTG-VBGE) acetone solution for 2 min, followed by rinsing with acetone and distilled water before drying with a stream of nitrogen. 

The stability of two primers against esterase was tested by challenging primed HA pellets with Pseudocholine esterase from equine serum (PCE, Product No. C7512, Sigma, Saint Louis, MO, USA). The PCE was dissolved in a saline solution containing 40 mM magnesium chloride and 99 mM of sodium chloride following the vendor’s instruction [35]. The concentration of PCE is 15 units/mL (equals 13.4 units/mg protein, the range of the PCE activity found in human saliva: 4–18 units/mg protein [36]) with the activity assay followed by the procedures previously described [37]. Each primed HA pellet was incubated in 5 mL of PCE solution for 24 h at 37 °C. In parallel, the control specimen was incubated for the same duration in the same amount of saline solution. After incubation, the specimen was rinsed three times with ultrapure water, followed by washing with a 0.5 wt% sodium dodecyl sulfate solution, then rinsed again three times with ultrapure water, and dried under a stream of nitrogen.

### 2.6. Contact Angle (CA) Measurements

A drop shape analyzer (DSA100, KRÜSS GmbH, Hamburg, Germany) was used to measure the CAs at room temperature. A drop of 2 µL ultrapure water (resistivity 18.3 MΩ∙cm) or 2 µL adhesive was deposited onto the substrate surface. The images of the sessile droplet were captured with a CCD (charge-coupled device) camera immediately after deposition on the substrate and analyzed with KRÜSS’ ADVANCE software to acquire water contact angle on adhesives. The water CA on HA was the average of 15 measurements. Water CA values on three substrates (original HA, NTG-GMA-treated HA, NTG-VBGE-treated HA) before and after the PCE challenge were measured.

Adhesives’ spreading on dentin was assessed from the CA changes on the acid-etched and primed dentin surface. CA was recorded starting from the deposition of the sessile droplet onto the substrate, for up to 5 min, to evaluate the adhesive spreading kinetics. For all the CA measurements on etched and primed dentin substrate, two randomly-located measurements were made for each resin composition per tooth. These measurements were repeated on three teeth. Consequently, the CA results of adhesive spreading on conditioned (etched and primed) dentin are the average of six measurements (2 measurements × 3 teeth) on conditional dentin substrate, and each homemade resin composition (B/H or U/V) was evaluated and compared on the same substrate (conditioned dentin) to minimize the impact of biological and morphological variation of dentin.

### 2.7. Fracture Surfaces Morphological Observation and Failure Mode Analysis

The failure modes were investigated by a light microscope and confirmed in a scanning electron microscope (SEM). All fractured specimens from SBS and μTBS tests were observed under a binocular microscope (OLYMPUS BX50, OLYMPUS, Tokyo, Japan) at 30–50× magnifications. For SEM analysis, the fracture surfaces were sputter-coated with gold before being observed under SEM (JSM-IT500, JEOL USA Inc., Peabody, MA, USA) equipped with a tungsten filament and a secondary electron detector. Secondary electron images with the 50–1000× magnifications were recorded under a high vacuum at an accelerating voltage of 5 kV. 

### 2.8. Statistical Analysis

The experimental results were analyzed using one-way analysis of variance (ANOVA) with a 95% confidence interval used to indicate significant differences (*p* < 0.05). The post-hoc Tukey HSD (honestly significant difference) tests were applied for ranking different specimen groups. The difference in failure mode was analyzed using the Chi-squared test and Fisher’s exact test. 

## 3. Results

### 3.1. Hydrophilicity of NTG-VBGE and Its Stability against PCE Challenge

In this study, the hydrophilicity of NTG-GMA and that of NTG-VBGE were compared by measuring the CA (θ) value of water droplets on hydroxyapatite (HA) crystals with surfaces modified with NTG-GMA and NTG-VBGE, respectively.

**Figure 2:**
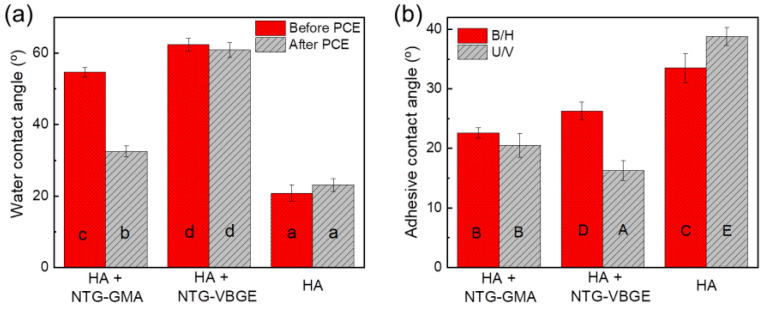
Contact angles (CA) of water (**a**) and adhesive (**b**) droplets on HA crystal and primed HA surfaces. Significant differences (*p* < 0.05) are indicated by different letters with CA values following the order a < b < c < d and A < B < C < D < E.

Compared to the methacrylate group, the vinylbenzene group is generally considered more hydrophobic [22]. Water contact angles measured on the HA crystals treated with two primers were 54.7 ± 1.3° for the NTG-GMA-treated surface and 62.4 ± 1.8° for the NTG-VBGE-treated surface (Figure 2a), both of which are higher than the value on the untreated HA surface (20.8 ± 2.3°). This confirmed the successful surface modification of the HA crystals as well as NTG-VBGE being more hydrophobic than NTG-GMA.

To confirm the stability of NTG-VBGE against hydrolysis by esterase, we use primed HA crystals and tested the stability of the surface modification against the PCE challenge. After the PCE incubation, the water CA on NTG-GMA-primed HD dropped to 32.5 ± 1.5°, while the CAs on NTG-VBGE remained unchanged. 

To understand the interaction between the adhesive resin and the two primers, we tested the wettability of adhesive on primed HA crystals by measuring the contact angles of resin droplets on primed HA crystal surfaces. As shown in Figure 2b, B/H adhesive has a smaller CA value on NTG-GMA-modified HA than that on NTG-VBGE-modified HA, indicating that B/H adhesive has a greater affinity to NTG-GMA than NTG-VBGE. On the other hand, U/V adhesive has a greater affinity to NTG-VBGE than NTG-GMA.

### 3.2. Dentin Bond Strength Evaluations

**Figure 3:**
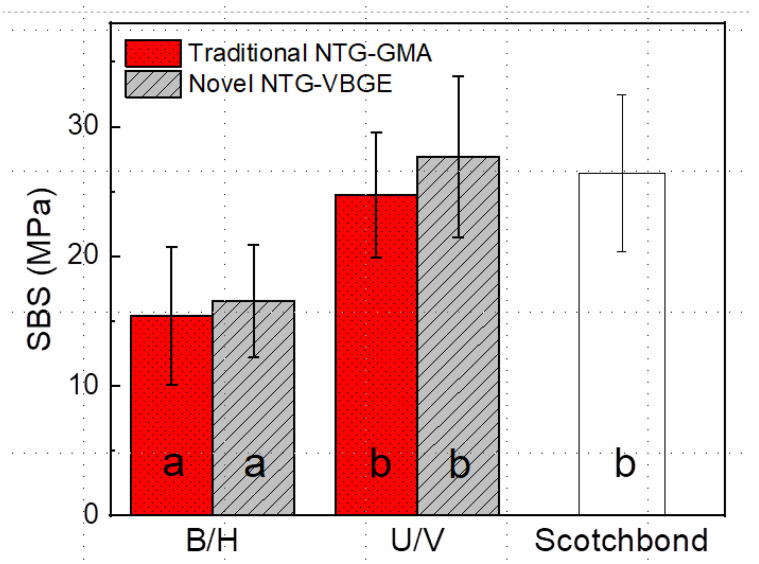
Shear bond strength (SBS) of dental adhesives with two primers. Significant differences (*p* < 0.05) are indicated by different letters with bond strength values following the order a < b.

Figure 3 shows the SBS results of four test groups as well as the control. U/V has equivalent SBS values as the control while they are higher than values of B/H adhesives using either of the two primers. The results are consistent with our previous study [32]. There is no significant effect of primer on SBS values of bonding for either B/H or U/V adhesives.

The μTBS results with 0-TC were listed in Table 1. The results are also shown in Figure S4 as a box plot. The NTG-VBGE + U/V group had the highest μTBS, while the other three groups, NTG-GMA + U/V, NTG-GMA + B/H, and NTG-VBGE + B/H, achieved the equivalent μTBS as the commercial control Scotchbond adhesive. 

### 3.3. Durability Evaluation by TC

As shown in Table 1, after 10,000-TC, two test groups containing B/H adhesive showed a significant drop in μTBS values: a 42% drop for the NTG-GMA + B/H group and a 20% drop for the NTG-VBGE + B/H group. Although here we observed a difference in the percentage of μTBS dropping, further experiments need to be conducted to determine whether NTG-VBGE can help improve the bonding durability of B/H adhesive. The commercial control Scotchbond adhesive also showed a decreased μTBS value after 10,000-TC. The results are consistent with our previous paper in which only traditional primer NTG-GMA was used [32]. Both testing groups containing U/V adhesive showed no drop in μTBS values, confirming the enhanced durability of U/V adhesive.

### 3.4. Failure Mode Analysis 

For SBS tests, mixed failures were observed on each fractured tooth/composite interface (Figure S5). For µTBS tests, three types of failure were observed: cohesive failure in the adhesive layer (C) with a fracture surface consisting of only adhesive; adhesive failure along the dentin surface (AD); and mixed failure (M) with fractures traveling into either composite or dentin. Some representative SEM images are shown in Figure S6. There was no pre-test failure, dentin failure, adhesive failure along the composite surface, or composite failure that occurred in this study. A summary of the failure mode can be found in Table 1.

For µTBS at both 0-TC and 10,000-TC, we found the highest frequency of type C failure (cohesive failure in the adhesive layer) in the NTG-VBGE+U/V group. As several studies supported that cohesive failures are related to a high bond strength [38,39], it is suggested that the NTG-VBGE+U/V group achieved the strongest bonding, which is consistent with the µTBS results shown in Figure 3. The NTG-GMA+B/H group and the NTG-VBGE+B/H group showed the highest number of type AD failures, which is consistent with the lower SBS values obtained (Table 1). 

If we compare the failure modes between 0-TC and 10,000-TC for each group, only the NTG-VBGE+U/V group showed no significant difference (*p* = 0.88). The other four groups showed a trend of an increased number of type AD failures and a decreasing trend in the number of type C failures, indicating a deteriorated dentin–adhesive bonding interface after TC [40].

### 3.5. Resin Spreading Kinetics

**Figure 4:**
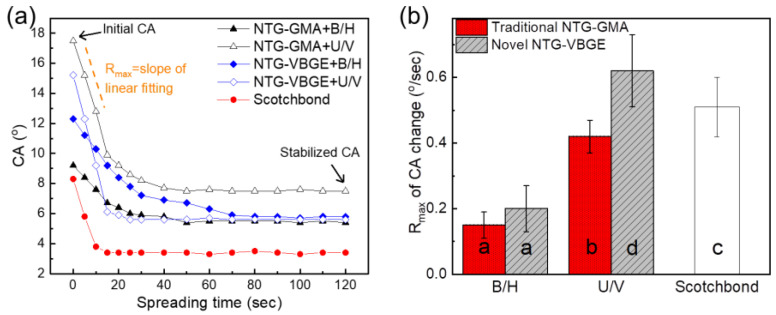
(**a**) Contact angle (CA) evaluation of adhesive spreading on primed dentin substrates; (**b**) R_max_ values calculated as the slope of linear fitting of the initial 15 s of resin spreading curves in (**a**). Significant differences (*p* < 0.05) in R_max_ values are indicated by different letters with values following the order a < b < c < d.

Figure 4a shows the resin spreading kinetics on conditioned dentin. Three parameters, which illustrate the trends discovered by the CA measurements, were highlighted in Table 1. These parameters are the initial CA, the stabilized CA, and the maximum rate of CA changes (R_max_) during the initial 15 s of interaction between the adhesives and the substrates (Figure 4a,b, Table 1). All adhesives formed a larger initial CA, then the CA decreased with adhesive infiltration, and then stabilized after 40 s. It is worth noting that the initial CA values here are significantly lower than the adhesive CA values on primed HA crystal surfaces in Figure 2b. The reason is that the priming of the dentin surface after removing the smear layer served to increase the surface free energy and to improve the wettability of the bonding agent on the dentin [41].

The initial CA captures the moment when the resin first interacted with the substrates. The stabilized CA indicated the end of resin infiltration. As adhesives’ infiltration into dentin tubules is mainly driven by capillary action [42,43], the R_max_, calculated from the slope of linear fitting of the initial CAs as a function of time, represents the spreading kinetics during the initial moments, such as spreading acceleration and culmination. 

The results showed that the NTG-VBGE group had the highest R_max_. Meanwhile, for the U/V adhesive, NTG-VBGE generated a 39% higher R_max_ than NTG-GMA did. However, for the B/H adhesive, the R_max_ values showed no significant difference between the two groups using NTG-GMA and NTG-VBGE, respectively. Similar to our previous study [32], the novel ether-based adhesive U/V had a much higher R_max_ than traditional B/H adhesive. Although the Scotchbond control adhesive is based on BisGMA/HEMA, the reason for its much higher R_max_ value than B/H is unknown, as the exact compositions of the Scotchbond adhesive and primer are trade secrets.

## 4. Discussion

Traditional three-step etch-and-rinse adhesives use primers containing hydrophilic monomers and solvents, aiming to displace water and prepare the collagen scaffold for the infiltration of the solvent-free, hydrophobic bonding resin [44]. Simplified two-step etch-and-rinse systems combine the hydrophilic primer and the hydrophobic resin into one solution. Self-etch systems contain acidic resin monomers that simultaneously etch and prime the dental substrate, and they are subdivided into two-step and one-step categories [45]. Despite the development of faster and more simple adhesives, conventional three-step and two-step self-etching adhesives are still considered the most reliable alternatives and the benchmark for dental adhesion [46]. Furthermore, in terms of new materials development, a three-step bonding procedure is the best way to identify the performance of the individual component. Therefore, the classical three-step etch-and-rinse bonding procedure was adopted in this study.

The loss of resin–dentin bond integrity and the reduction in bond strength were attributed partly to the hydrophilic nature of the contemporary adhesives systems that causes unwanted water absorption, phase separation, and resin leaching, and also to the endogenous collagenolytic enzymes that can slowly hydrolyze collagen [14]. Current research in this field aims at increasing the durability of resin–dentin bonds by inhibiting the collagenolytic activity of dentin, as well as implementing bonding strategies that allow the use of more hydrophobic bonding agents [14]. As reported in our previous publication [32], the new ether-based adhesive U/V is more hydrophobic than traditional methacrylate-based adhesive B/H, and U/V achieved an enhanced durability as well as a reduced water sorption/solubility. Here, we confirmed that the NTG-VBGE is a more hydrophobic primer than traditional NTG-GMA. Together with the nonhydrolytic nature of the ether group (proved by PCE challenged of primed HA crystal, NTG-VBGE primer can be a step forward toward developing more hydrophobic and more durable bonding systems. Therefore, the first hypothesis of this study, in which NTG-VBGE is more hydrophobic and more hydrolytically stable than NTG-DMA, has been shown.

The contact angle (CA), which is the internal angle between a liquid and a substrate, can be used to evaluate the interaction between the liquid and the substrate [47]. Generally, smaller CAs are achieved when the liquid has a greater affinity to the substrate. If the liquid is water, a smaller θ indicates that the substrate is more hydrophilic, while a larger θ indicates that the substrate is more hydrophobic. On the other hand, enamel and dentin layers in the tooth’s structure are predominantly composed of HA crystals [48]. Therefore, hydroxyapatite crystals have been used as versatile model surfaces that mimic enamel in their performance concerning bacterial adhesion [49], remineralization/demineralization [50], tooth staining [51], and other properties [52]. Although dentin only contains ~ 70wt% of HA, HA crystals were used in this study to confirm that primers can chemically bond to HA (and presumably the tooth surface), to compare the hydrophilicity of two primers, and to test the stability of the bonded primers against esterase challenge.

This study evaluated the bonding performance of two primers combined with two adhesive formulae, respectively. That means the new ether-based NTG-VBGE is compatible with both traditional B/H adhesive and novel U/V adhesive. The second hypothesis of this study is that NTG-VBGE can achieve strong dentin bonding with either traditional methacrylate-based or new ether-based adhesives. This hypothesis has been partially supported. The results showed that NTG-VBGE can achieve a higher μTBS with U/V adhesive than NTG-GMA, but not with B/H adhesive, probably because NTG-VBGE has a greater affinity with U/V as confirmed by adhesive CA measurements in Figure 2b. The reason why the NTG-VBGE+U/V group did not have a higher SBS than the NTG-GMA+U/V group may be the limitation of the SBS test [53,54,55]. The SBS data in the current experimental design are governed by the cohesive strength of the base materials used and not by the bond strength of the adhesive interface [53]. Some studies suggest that μTBS tests are more constant for a wide range of specimen shapes and testing setups than the SBS tests [55].

TC has been used to accelerate the fatigue of bonding. Alternating temperature between 5 °C and 55 °C simulates extreme cold and warm oral environments [34]. Such a large temperature fluctuation also induces the shrinking and swelling of materials. In general, dental adhesives have a larger volume variation in response to temperature changes due to their low filler content in comparison with composites and dentin. Consequently, the bonding failures are more likely to take place within the dental adhesives or at the dentin/adhesive interface.

Upon challenge with TC, ether-based U/V adhesive has a better durability than the B/H adhesive as well as the BisGMA/HEMA-based commercial control, which is consistent with the results of our previous study [32]. Here, we found that there is no significant difference between the group using NTG-GMA and that using NTG-VBGE as a primer. The possible reason is that TC, through the temperature parameter, simulates the entrance of hot and cold substances in the oral cavity, and shows the relationship of the linear coefficient of thermal expansion between the tooth and the restorative material [56]. As the primer layer is much thinner than the adhesive layer, the effect of thermal expansion and contraction could be negligible. Other durability testing methods, such as enzymatic/bacterial challenge and long-term storage, are needed to prove the effectiveness of NTG-VBGE on the durability of the restoration. 

Our previous study showed that U/V resin is more hydrophobic than B/H resin [32], and the results showed that NTG-VBGE is more hydrophobic than NTG-GMA (Figure 2a). A more direct way to understand the interaction between the adhesive resin and a substrate is to directly measure the contact angle of the adhesive droplet on the substrate. We also assessed adhesive spreading on conditioned dentin surfaces using real-time CA measurement. After acid etching, the smear layer was removed, and the collagen was exposed. The primer step resulted in an additional layer of primer, composed of NTG-GMA/NTG-VBGE, PMGDM, and HEMA (composed of Scotchbond primer in the case of Scotchbond adhesive), on the dentin surface. We applied these conditioned dentin substrates to evaluate the adhesives’ interactions with exposed collagen and primer-coated surfaces. Specifically, the primed dentin duplicates the exact conditions in applying dental adhesives for SBS and μTBS tests. 

In this study, we observed a larger contact angle of the U/V resin droplet on NTG-GMA-treated HA crystals than that on NTG-VBGE-treated HA crystals (Figure 2b). In addition, the initial contact angle of the U/V droplet on dentin primed by NTG-GMA is higher than the value on dentin primed with NTG-VBGE. As a smaller contact angle indicates a greater affinity between the liquid and the substrate, U/V adhesive has a greater affinity with NTG-VBGE-primed dentin. As a result, the NTG-VBGE primer created a better dentin wettability and infiltration for the more hydrophobic U/V adhesive, as indicated by the results that the NTG-VBGE+U/V group had the highest R_max_ (Figure 4b and Table 1). As a whole, the third hypothesis of this study, in which NTG-VBGE can facilitate a better dentin filtration for ether-based adhesive, has been shown.

## 5. Conclusions

The ether-based primer NTG-VBGE is compatible with both the traditional methacrylate-based adhesive B/H and the ether-based adhesive U/V to achieve SBS and μTBS values either equivalent to or higher than those of the commercial control. Moreover, their bonding stability exceeded that of the commercial control under extended challenges by TC. The NTG-VBGE+U/V group has the highest μTBS. Furthermore, the superior adhesive performance correlated well with the enhanced dentin spreading (infiltration) kinetics. The increased hydrophobicity and the hydrolytic stability of NTG-VBGE and U/V adhesive suggest significant potential for further developing dental restorative materials with an extended service life.

## Figures and Tables

**Table 1:** Results of microtensile bond strength tests combined with TC and failure mode analysis.

	0-TC		10,000-TC	
Group	Mean (MPa)	Number of Failures by Mode (C/AD/M)	Mean (MPa)	Number of Failures by Mode (C/AD/M)
Scotchbond	40.4 ± 8.4 ^a^	9/1/5	28.4 ± 11.6 *	6/7/2 **
NTG-GMA + B/H	39.3 ± 12.3 ^a^	7/8/0	22.8 ± 14.0 *	4/10/1 **
NTG-GMA + U/V	38.5 ± 8.7 ^a^	9/3/3	41.4 ± 13.4	7/7/1 **
NTG-VBGE + B/H	34.4 ± 11.5 ^a^	8/7/0	26.4 ± 8.4 *	5/10/0 **
NTG-VBGE + U/V	48.7 ± 12.4 ^b^	11/2/2	46.4 ± 12.1	10/2/3

* Note. Means (MPa) ± standard deviation of microtensile bond strength values. Significant differences (*p* < 0.05) for µTBS at 0-TC are indicated by different superscript letters with bond strength values following the order a < b. Mean µTBS values and failure modes stastiscs of 10,000-TC with “*” and “**” show the values are statistically different from the values of 0-TC in the same row. Failure mode: C = cohesive failure in the adhesive layer; AD = adhesive failure along the dentin surface; M = mixed failure.

## Data Availability

The datasets generated during and/or analyzed during the current study are available from the corresponding author on reasonable request.

## Supplementary Materials

The following supporting information can be downloaded at: https://www.mdpi.com/2079-4983/13/3/128/s1, Table S1: Summary of resin compositions and their functions in dental restorations; Figure S1: Illustration of bonding procedures; Figure S2: Illustration of procedures for shear bond strength (SBS) evaluation; Figure S3: Illustration of procedures for micro-tensile bond strength (μTBS) evaluation and thermal cycling (TC); Figure S4: Microtensile bond strength (μTBS) values of five testing groups; Figure S5: Representative fractured SBS specimens under optical microscopy; Figure S6: Representative fractured μTBS specimens under scanning electron microscopy.
