# Correction: Wang et al. Improve Dentin Bonding Performance Using a Hydrolytically Stable, Ether-Based Primer. *J. Funct. Biomater.* 2022, *13*, 128

**DOI:** 10.3390/jfb14080419

**Published:** 2023-08-09

**Authors:** Xiaohong Wang, Shinobu Yamauchi, Jirun Sun

**Affiliations:** 1 American Dental Association Science & Research Institute, Gaithersburg, MD 20899, USA; yamauchis0506@gmail.com; 2 Research Center for Electron Photon Science, Tohoku University, Sendai 982-0826, Japan; 3 The Forsyth Institute, Cambridge, MA 02142, USA; jsun@forsyth.org; 4 Harvard School of Dental Medicine, Boston, MA 02115, USA

## Addition of an Author

Jirun Sun was not included as an author in the original publication [1]. The corrected Author Contributions statement appears here. 

**Author Contributions:** Conceptualization, X.W., S.Y. and J.S.; methodology, S.Y. and J.S.; validation, X.W.; formal analysis, X.W.; investigation, X.W.; resources, X.W. and J.S.; data curation, X.W.; writing—original draft preparation, X.W.; writing—review and editing, X.W. and S.Y.; visualization, X.W. All authors have read and agreed to the published version of the manuscript.

## Missing Funding

In the original publication, the funder, NIH, grant number DE023752 to Jirun Sun, was not included. The updated funding section appears here. 

**Funding:** This research was funded by NIH, grant number DE023752.

## Additional Affiliations

In addition to affiliations 1 and 2, the updated affiliations should include the following:

^3^ The Forsyth Institute, Cambridge, MA 02142, USA

^4^ Harvard School of Dental Medicine, Boston, MA 02115, USA

The authors state that the scientific conclusions are unaffected. This correction was approved by the Academic Editor. The original publication has also been updated.
