# Supplementary material for: Improve Dentin Bonding Performance Using a Hydrolytically Stable, Ether-Based Primer
Source: J Funct Biomater. 2022 Aug 26;13(3):128. doi: 10.3390/jfb13030128 (PMC9501844; doi:10.3390/jfb13030128)
Supplement: Supplementary file 1 [file jfb-13-00128-s001.zip › jfb-1834221-supplementary.pdf]

Supplementary Materials

Improve Dentin Bonding Performance Using a Hydrolytically Stable, Ether-Based Primer

Xiaohong Wang [1,*] and Shinobu Yamauchi [1,2]

[1] American Dental Association Science & Research Institute, Gaithersburg, MD 20899, USA
[2] Research Center for Electron Photon Science, Tohoku University, Sendai, Miyagi 982-0826, Japan
* Correspondence: wangx@ada.org

Contents:

Table S1. Summary of resin compositions and their functions in dental restorations
Figure S1. Illustration of bonding procedures
Figure S2: Illustration of procedures for shear bond strength (SBS) evaluation
Figure S3: Illustration of procedures for micro-tensile bond strength (μTBS) evaluation and thermal cycling (TC)
Figure S4: Microtensile bond strength (μTBS) values of five testing groups
Figure S5. Representative fractured SBS specimens under optical microscopy
Figure S6. Representative fractured μTBS specimens under scanning electron microscopy.

Table S1. Summary of resin compositions and their functions in dental restorations*

Acronym	Composition	Detail composition	Primer	Adhesive	Composite
NTG-GMA	NTG-GMA<br>PMGDM+ HEMA	5% NTG-GMA acetone solution<br>20% PMGDM+ HEMA 1:1 CQ/4EDMAB acetone solution	Y		
NTG-VBGE	NTG-VBGE<br>PMGDM+ HEMA	5% NTG-VBGE acetone solution<br>20% PMGDM+ HEMA 1:1 CQ/4EDMAB acetone solution	Y		
B/H	BisGMA/ HEMA	BisGMA/ HEMA 60/40 by weight, CQ/4EDMAB		Y	
U/V	UDMA/TEG-DVBE	UDMA/TEG-DVBE (1:1 mole), CQ/4EDMAB		Y	
Z250	Filtek™ Z250 Universal Restorative (3M ESPE, St. Paul, MN, USA)				Y
Scotchbond	Adper™ Scotchbond™ Multi-Purpose Adhesive (3M ESPE, St. Paul, MN, USA)		Y	Y	

*Note: Y indicates that the corresponding resin is used in a primer, adhesive, or composite. Abbreviations of chemicals in the table: 2-bis(4-(2-hydroxy-3-methacryl-oxypropoxy)-phenyl)-propane) (Bis-GMA), urethane dimethacrylate (UDMA), 2-hydroxyethyl-methacrylate (HEMA), pyromellitic glycerol dimethacrylate (PMGDM), triethylene glycol divinylbenzyl ether (TEG-DVBE), glycine, N-(2-hydroxy-3-(2-methyl-1-oxo-2-propenyl)propyl)-N-(4-methylphenyl), monosodium salt (NTG-GMA), glycine, N-2-hydroxy-3-(4-vinylbenzyloxy)-propyl-N-(4-methylphenyl), monosodium salt (NTG-VBGE), camphorquinone (CQ), ethyl 4-N, N-dimethylaminobenzoate (4EDMAB).

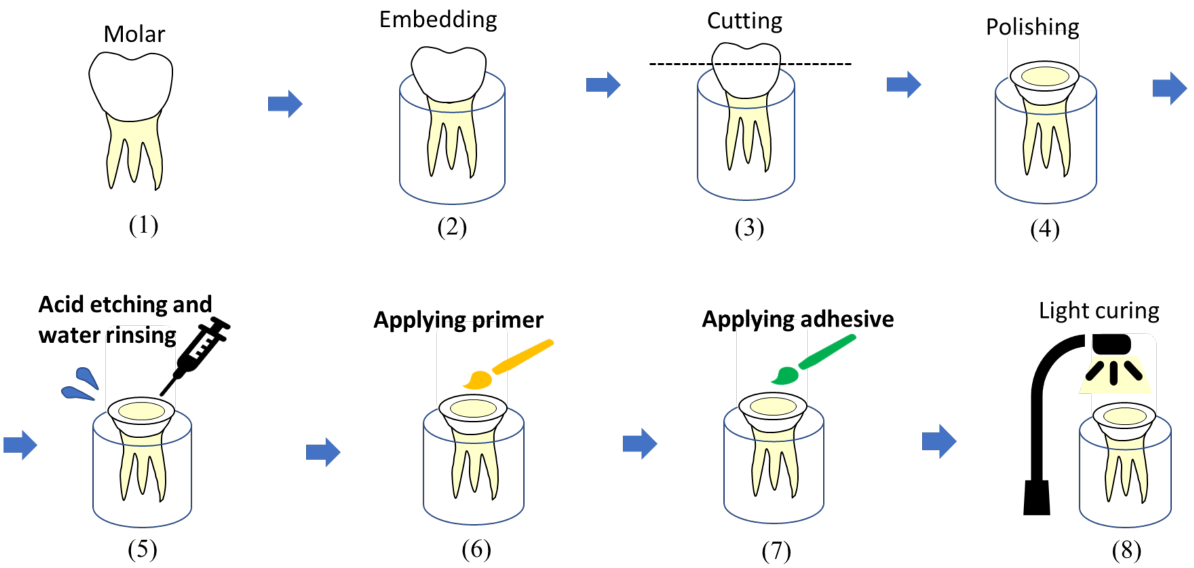

Figure S1. Illustration of procedures of tooth embedding and 3-step bonding procedures. We use the classical 3-step bonding procedure. After a molar (1) was embedded (2), the enamel part was cut (3) and polished (4), the dentin surface was acid-etched (5), apply with primer (6), and adhesive (7), then light-cured (8).

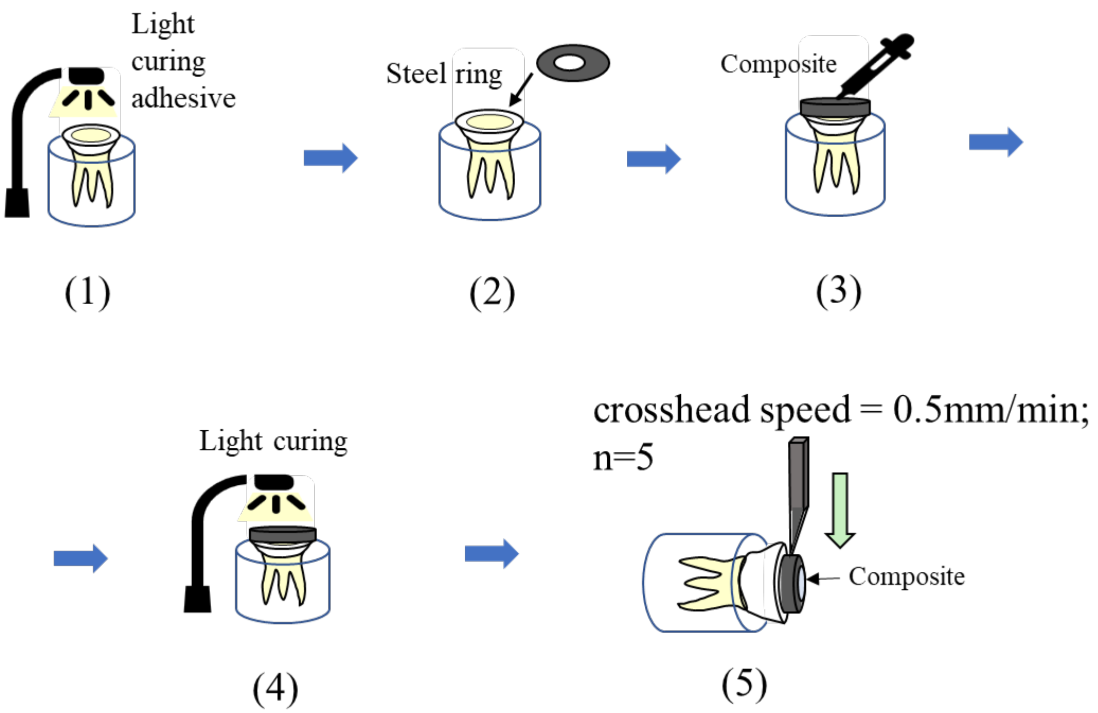

Figure S2. Illustration of procedures for shear bond strength evaluation. After curing adhesive (1), a metal ring was applied on top of the dentin (2), and the composite was applied within the ring (3). After curing composites (4), the sample was broken by a shear force for SBS evaluation (5).

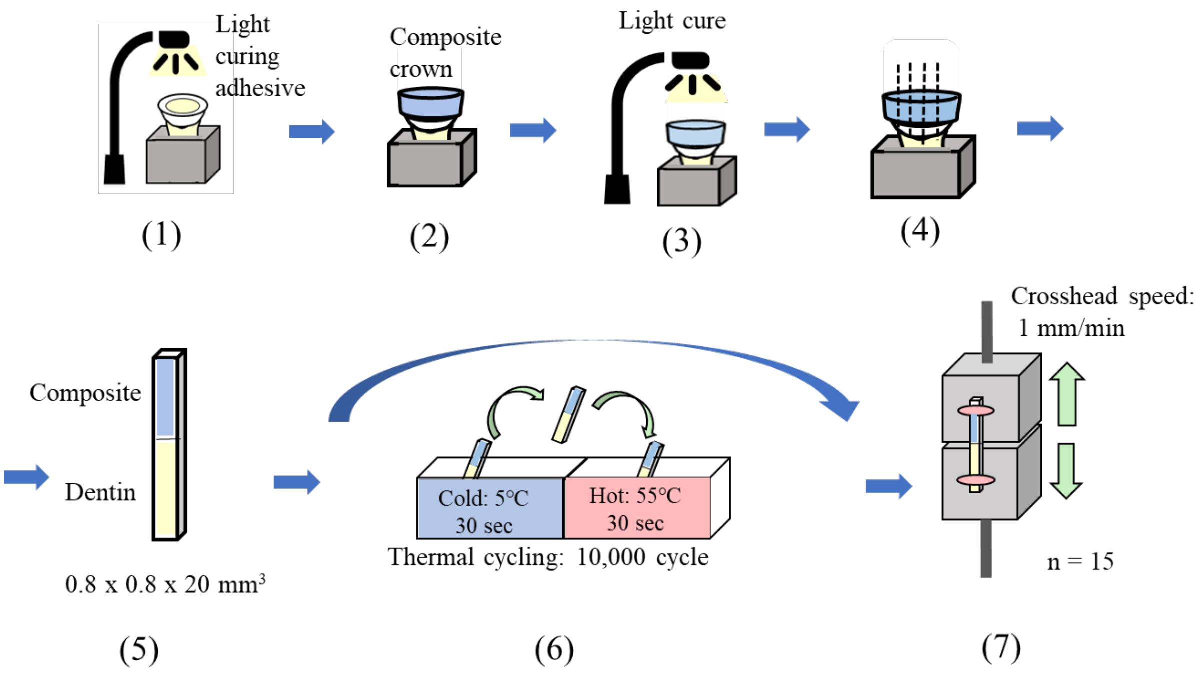

Figure S3. Illustration of procedures for micro-tensile bond strength (μTBS) evaluation and thermal cycling (TC). After curing adhesive (1), a composite crown was built (2) and cured (3). Then the embedded tooth was sliced (4) into beams (5). The beams were undergoing 0 or 10,000 cycles of thermal cycling (6) before μTBS tests (7).

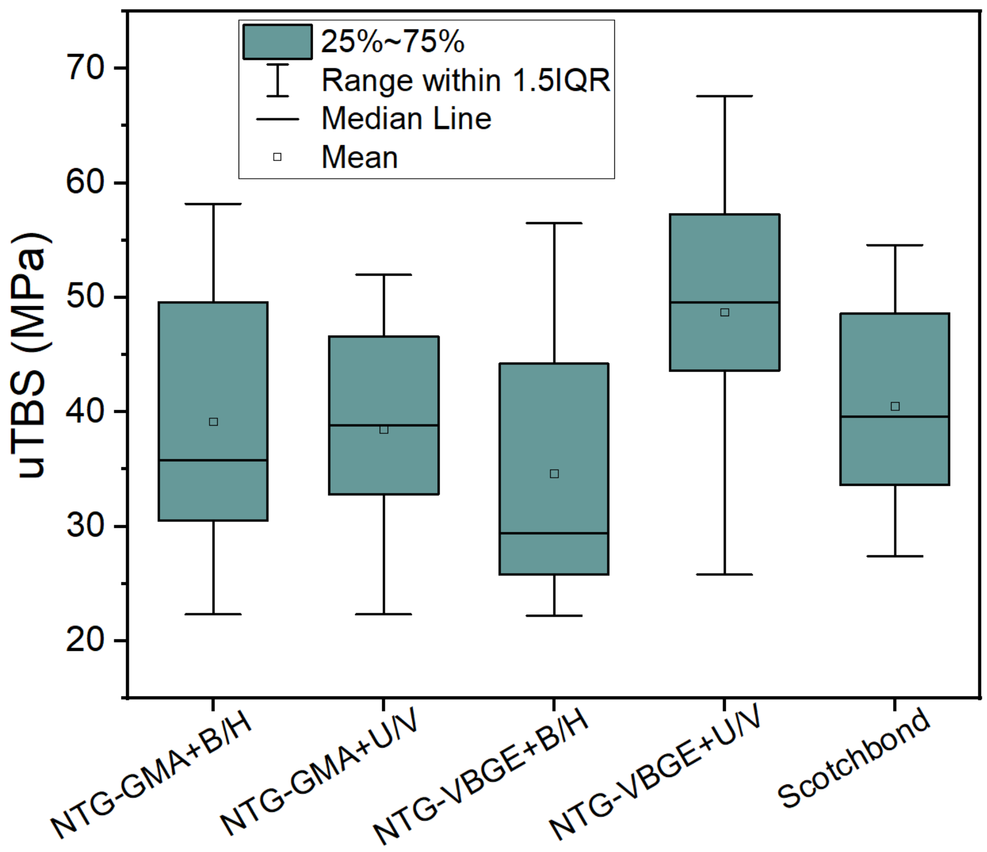

Figure S4. Microtensile bond strength (μTBS) values of five testing groups before thermal cycling. There is no outlier in the box plot.

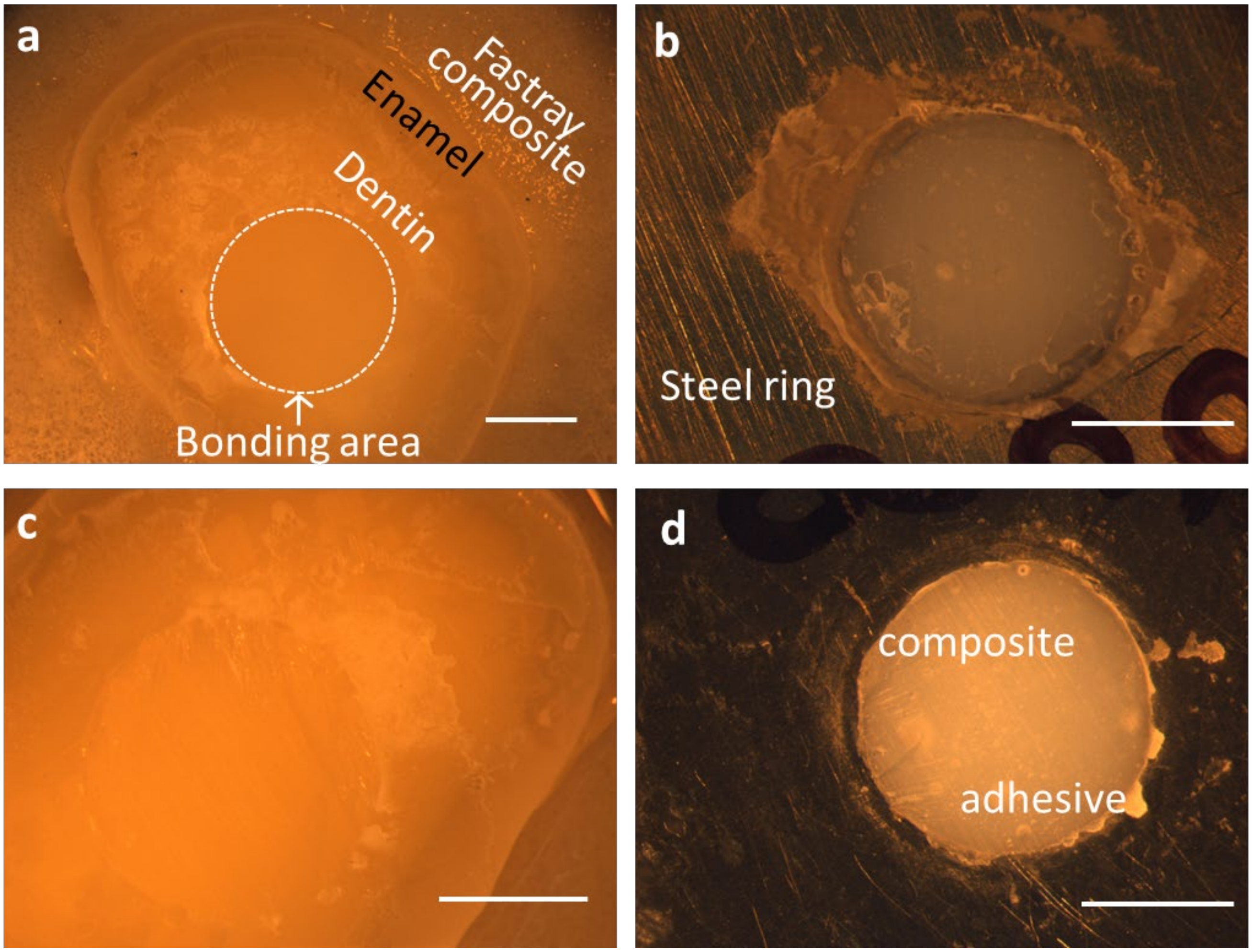

Figure S5. Representative fractured SBS specimens under optical microscopy: interfaces at the tooth side (a & c) and the composite side (b & d). Scale bars: 2 mm.

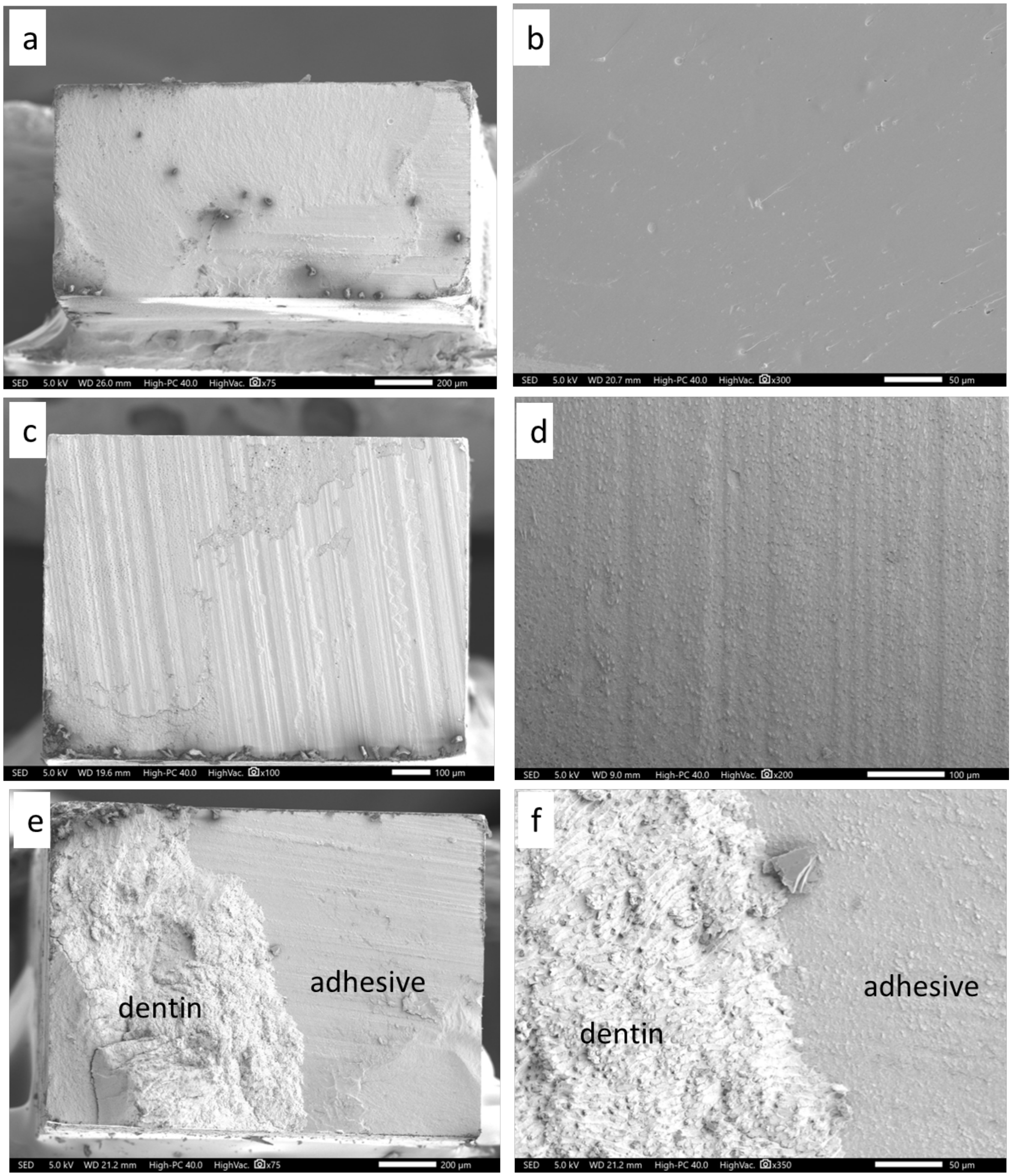

Figure S6. Representative fractured μTBS specimens under scanning electron microscopy. (a) & (b): cohesive failure in the adhesive layer; (c) & (d): adhesive failure along the dentin surface; (e) & (f): mixed failure.
